# Bio-Inspired Mamba for Antibody–Antigen Interaction Prediction

**DOI:** 10.3390/biom15060764

**Published:** 2025-05-26

**Authors:** Xuan Liu, Haitao Fu, Yuqing Yang, Jian Zhang

**Affiliations:** 1 School of Computer and Information Technology, Xinyang Normal University, Xinyang 464000, China; lx666@xynu.edu.cn; 2 School of Artificial Intelligence, Hubei University, Wuhan 430062, China; fuhaitao@hubu.edu.cn; 3 School of Management, Xinyang Agriculture and Forestry University, Xinyang 464000, China; 2017220006@xyafu.edu.cn

**Keywords:** antibody–antigen interaction prediction, deep learning, protein language model

## Abstract

Antibody lead discovery, crucial for immunotherapy development, requires identifying candidates with potent binding affinities to target antigens. Recent advances in protein language models have opened promising avenues to tackle this challenge by predicting antibody–antigen interactions (AAIs). Despite their appeals, precisely detecting binding sites (i.e., paratopes and epitopes) within the complex landscape of long-sequence biomolecules remains challenging. Herein, we propose MambaAAI, a bio-inspired model built upon the Mamba architecture, designed to predict AAIs and identify binding sites through selective attention mechanisms. Technically, we employ ESM-2, a pre-trained protein language model to extract evolutionarily enriched representations from input antigen and antibody sequences, which are modeled as residue-level interaction matrixes. Subsequently, a dual-view Mamba encoder is devised to capture important binding patterns, by dynamically learning embeddings of interaction matrixes from both antibody and antigen perspectives. Finally, the learned embeddings are decoded using a multilayer perceptron to output interaction probabilities. MambaAAI provides a unique advantage, relative to prior techniques, in dynamically selecting bio-enhancing residue sites that contribute to AAI prediction. We evaluate MambaAAI on two large-scale antibody–antigen neutralization datasets, and in silico results demonstrate that our method marginally outperforms the state-of-the-art baselines in terms of prediction accuracy, while maintaining robust generalization to unseen antibodies and antigens. In further analysis of the selective attention mechanism, we found that MambaAAI successfully uncovers critical epitope and paratope regions in the SARS-CoV-2 antibody examples. It is believed that MambaAAI holds great potential to discover lead candidates targeting specific antigens at a lower burden.

## 1. Introduction

Antibodies have emerged as successful clinical therapeutics for a wide range of human diseases, which is attributed to their capabilities to bind target antigens with favorable properties like high affinity and specificity [1,2]. At present, there are nearly 1200 therapeutic antibodies in clinical studies and over 170 that are approved or undergoing regulatory review [3]. Therapeutic antibody development typically begins with lead discovery, involving the identification of interactions (e.g., binding affinity, neutralization) between candidate antibodies and target antigens [4]. However, traditional bioassay experiments based on animal immunization in vivo [5] or phage/yeast-surface display libraries in vitro [6] tend to be time-consuming and costly, thus requiring high-throughput computational methods to efficiently determine whether an antibody and antigen can interact, that is, antibody–antigen interaction (AAI) prediction.

Recently, artificial intelligence applied to AAI prediction is a pivotal topic, with the goal of speeding up antibody discovery by training models on known interaction data to predict unknown ones [7]. Current computational methods for modeling AAIs fall into two major categories. One category is the structure-based methods [8,9,10], which are devoted to using geometric deep learning to extract spatial features from 3D structures or complexes, particularly in the binding interfaces like complementarity determining regions (CDRs) [11]. Schneider et al. [12] implemented a convolutional neural network (CNN) for AAI predictions, which is trained on rigid-body docking poses (generated by the ZDOCK surrogate) of antibody structures in complex with antigen epitopes. Pittal et al. [13] constructed a graph neural network (GNN) capable of learning context-aware structural representations for both antigens and antibodies, so as to predict their binding interfaces. Gao et al. [14] leveraged informed AlphaFold2 [15] to infer AAIs based on the confidence of complex structure modeling. Yet, their performance often suffers from limited 3D data, with model training relying on generative tools to compensate for missing structural information. In contrast, sequence-based methods (another category) [16,17,18], which utilize large-scale sequence data, have seen considerable progress. Jin et al. [19] integrated sequential characteristics of antibodies/antigens into a unified deep-learning framework, and employed the Transformer model to capture the intrinsic dynamics of AAIs. Zhang et al. [20] also developed a GNN to enhance the prediction of neutralization activities by capturing molecular similarities between antibodies and antigens. Nowadays, pre-trained protein language models (PPLMs) such as ESM [21] and Ablang [22], have further advanced AAI prediction [23]. Yuan et al. [24] investigated two PPLMs (TAPE [25] and Ablang) to transform antigen and antibody sequences into pre-trained embedding vectors, followed by a CNN-based classifier to predict their affinities. Xia et al. [26] made use of multiple sequence information from PPLMs, to explore the interaction mechanisms of AAIs thoroughly. With these efforts, many methods have increasingly focused on developing attention mechanisms that can efficiently process AAI prediction while prioritizing binding sites, such as paratopes and epitopes [13,27].

In practice, the variable region of antibodies typically spans 200 amino acids (or residues), while antigens (e.g., viral surface proteins) can exceed 800 residues [28]. Such long sequences significantly exacerbate the computational burden of self- or cross-attention models, as each residue (named token) must compute attention weights with all others to establish a global relationship [27,29]. Beyond that, antibody–antigen binding is predominantly dictated by a small subset of critical residues, particularly those in the paratopes of antibodies and the epitopes of antigens [11], while other sequence regions contribute minimally. For instance, in SARS-CoV-2, the epitopes on the spike protein dominate immune recognition and neutralization [30]. Therefore, a major challenge lies in efficiently focusing on these key residue sites while filtering out irrelevant ones. In this context, selective state space models (SSMs) [31,32,33] have demonstrated great potential in handling long sequence data, thanks to convolutional and near-linear computations. Among them, Mamba [34], a structured SSM, emerges as a compelling alternative to Transformers by capturing long-range dependencies more effectively. For example, Zhu et al. [35] extended Mamba to the computer vision tasks, by incorporating bidirectional SSMs for global visual context modeling and position embeddings for location-aware understanding. Ali et al. [36] reformulated Mamba computation with a data-control linear operator, which not only exposed hidden attention matrices but also facilitated the application of established interpretability methods to identify critical tokens. Motivated by these advancements, we believe that Mamba could offer a promising solution for dynamically concentrating on relevant residues in AAI prediction. Nevertheless, how to incorporate the Mamba architecture into this field remains a challenge.

In pursuit of this goal, we propose a bio-inspired Mamba model for the antibody–antigen interaction prediction, namely MambaAAI. Specifically, we leverage ESM-2, a pre-trained protein language model, to extract evolutionarily enriched representations from input antigen and antibody sequences. Instead of modeling at the whole antigen or antibody level, we decompose each AAI instance into pairwise residue interactions and quantify them using a bilinear function, yielding an interaction matrix. Next, we devise a dual-view Mamba encoder to capture key binding patterns, which dynamically learns embeddings of interaction matrixes from both horizontal (antibody) and vertical (antigen) perspectives. Empowered by Mamba’s SSM mechanism, this dual-view strategy can enhance embedding learning of binding sites (i.e., paratopes and epitopes) while filtering out irrelevant sites. Finally, the learned embeddings are decoded through a multilayer perceptron to predict interaction probabilities of input antibody–antigen pairs. In the computational experiments on two antibody–antigen neutralization datasets, HIV and CoV-AbDab, MambaAAI marginally outperformed existing methods in terms of prediction accuracy, and offered superior generalization to unseen antigen-antibody pairs. Through end-to-end training, the selective attention behind Mamba process can be regarded as a traceable path to explicitly highlight which residue sites contribute more to their interaction outcome, thus helping to understand the AAI predictions. In downstream case studies about SARS-CoV-2 antibodies, we found that MambaAAI not only successfully pinpoints critical epitopes and paratopes, but also exhibits strong potential as a powerful tool for screening antibody candidates.

## 2. Materials and Methods

### 2.1. Data Preparation

In this study, the model training and testing for AAI predictions were performed on two large-scale antibody–antigen (Ab-Ag) neutralization databases: HIV [37] and CoV-AbDab [38]. Details of both databases are as follows:

**HIV database** included a vast collection of neutralization antibodies associated with the human immunodeficiency virus. In accordance with the protocol established by [20], we filtered out Ab-Ag pairs with homology exceeding 0.9 for both antigens and antibodies. Finally, we compiled an HIV dataset containing 24,907 neutralization pairs (positive samples) and 26,480 non-neutralization pairs (negative samples, random sampling from remaining combinatorial space), which encompassed 1752 antigens and 457 antibodies. Here, antigen sequences ranged from 649 to 912 residues, while antibody sequences represented full-length heavy chains, ranging from 98 to 462 residues.

**CoV-AbDab database** provided detailed information on conventional antibodies or nanobodies capable of binding to various coronaviruses. Since the CoV-AbDab includes only antigen names but not sequences, we retrieved antigen sequences with annotations for the receptor-binding domain (RBD) from the database’s provided references. Following the study [26], we intercepted the RBD region to serve as the antigen sequence, and collected the Ag-Ab neutralization and non-neutralization pairs. The final dataset consisted of 14,593 samples (5486 neutralization pairs and 9110 non-neutralization pairs) derived from 36 antigens and 4248 antibodies. Here, antigen sequences ranged from 183 to 305 residues, while antibody sequences (heavy chains) ranged from 100 to 226 residues.

### 2.2. Model Architecture

**Figure 1:**
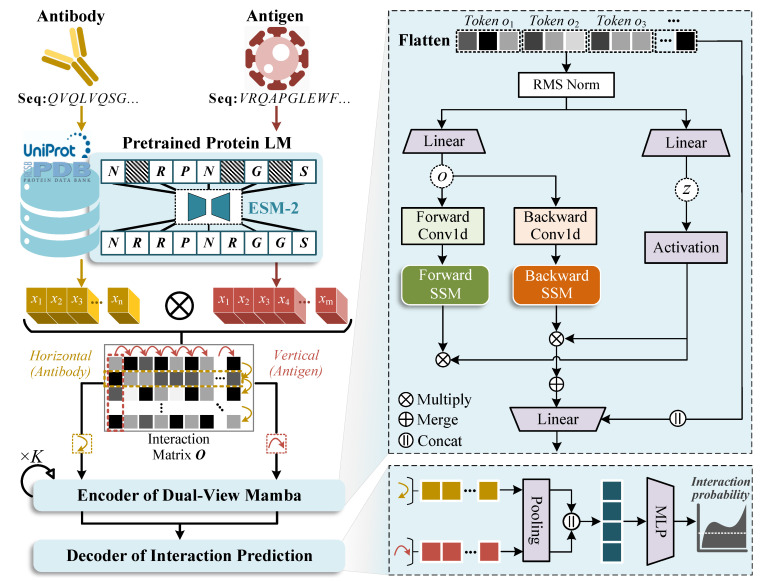
The model architecture of MambaAAI, which comprises three modules. (1) Representation extraction with PPLMs: informed representations of antibody and antigen sequences are extracted from a pre-trained protein language model ESM-2, and then structured into a residue-level interaction matrix. (2) Encoder of dual-view Mamba: the interaction matrix is split into horizontal and vertical patches, which are processed by the bidirectional SSM block to dynamically capture embeddings of critical binding sites. (3) Decoder of interaction prediction: the learned embeddings are aggregated via a pooling layer and fused into an MLP decoder to output AAI probabilities.

In our AAI prediction setting, we define a set of antibody sequences $A_{b}$, a set of antigen sequences $A_{g}$, and their interactions $I\in {\left\{0,1\right\}}^{|A_{b}\left|\times \right|A_{g}|}$, where $I_{i,j}=1$ if antibody *i* is annotated to interact with antigen *j*; otherwise, $I_{i,j}=0$. Given an antibody sequence $b_{i}\in A_{b}$ and an antigen sequence $g_{i}\in A_{g}$, the objective is to learn a mapping function that outputs an interaction probability score p∈[0,1], where a higher score indicates a greater likelihood of interaction. To this end, we develop the MambaAAI model (illustrated in Figure 1), which involves the following modules in an end-to-end manner. (1) First, given an antibody–antigen sequence pair, we extract their representation vectors using pre-trained protein language models (PPLMs). (2) Then, we feed these representations into a dual-view Mamba encoder to learn informative latent embeddings. (3) Finally, we deploy a neural network decoder to map embeddings into an interaction probability for the given antibody–antigen pair. In what follows, we elaborate on the above three modules.

#### 2.2.1. Representation Extraction with PPLMs

Extracting informed representations from antibody and antigen sequences can reduce input noise and provide robust knowledge for downstream prediction tasks. To do so, we bring in the ESM-2, i.e., a Transformer-based protein language model pre-trained on the UniRef50 database, whose representations embedded evolutionary information about large-scale protein sequences in nature. Specifically, given an input antibody/antigen sequence $\left[s_{1},s_{2},\ldots \right]$, where $s_{i}$ stands for an amino acid (or residue), it is first tokenized into a set of predefined alphabet: $T=\left[t_{1},t_{2},\ldots \right],t_{i}=\mathrm{token}\left(s_{i}\right)$.

Next, the tokenized sequence T is passed through multiple Transformer encoder layers, in which self-attention mechanisms generate context-aware representations at both residue and sequence levels, as follows:

(1) $$X_{Ab}\leftarrow \mathrm{Tranfomer}\left(T_{Ab}\right),\;X_{Ag}\leftarrow \mathrm{Tranfomer}\left(T_{Ag}\right)$$

where $X_{Ab}={\left[x_{i}\right]}_{i=1}^{n}$ (or $X_{Ag}={\left[x_{i}\right]}_{i=1}^{m}$) is the output representations of *n* (or *m*) residues for an antibody (or antigen) sequence, where $x\in R^{d}$ is a *d*-dimensional vector.

On that basis, we measure the pairwise interactions between antibody residues and antigen residues using the extracted representations. Technically, we devise an interaction function Ω with a simple bilinear scoring:

(2) $$\Omega :O\in R^{n\times m}\leftarrow X_{Ab}\cdot W\cdot X_{Ag}^{T}$$

where $W\in R^{d\times d}$ is a trainable parameter matrix, and the function Ω output is a two-dimensional interaction matrix O. However, further modeling and encoding this matrix is challenging: CNNs are limited by their locally receptive fields, while Transformers tend to diffuse attention uniformly across all residue pairs.

#### 2.2.2. Encoder of Dual-View Mamba

To handle the interaction matrix O properly, we design a dual-view Mamba encoder inspired by [35]. Initially, O is converted into two flattened 1D patches ($O^{h}$ and $O^{v}$) by partitioning it along the horizontal and vertical (i.e., row and column) axes.

(3) $$O^{h}=\left[o_{1}^{h},\ldots ,o_{n}^{h}\right],\;O^{v}=\left[o_{1}^{v},\ldots ,o_{m}^{v}\right]$$

where $o_{i}^{h}\in R^{m}=O\left[i,:\right]$ and $o_{j}^{v}\in R^{n}=O\left[:,j\right]$ can be treated as the enhanced residue tokens. This partitioning strategy is intuitive: horizontal or vertical views correspond to the antibody and antigen perspectives, which facilitates a focused representation of key binding sites (paratopes and epitopes). Meanwhile, interaction matrix O can be seamlessly adapted with the Mamba architecture, despite its original design for 1D sequences. Subsequently, we apply a linear projection layer to transform these two sequence patches into embeddings with positions, formulated as follows:

(4) $${\hat{O}}^{h}=O^{h}W^{h}+E_{pos}^{h},\;{\hat{O}}^{v}=O^{v}W^{v}+E_{pos}^{v}$$

where $E_{pos}$ is defined as the position embeddings of residue order, and $W^{h}$ and $W^{v}$ are the learnable projection matrixes.

**Figure 2:**
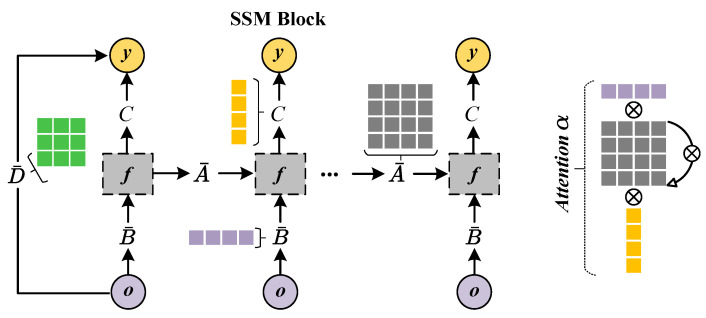
Pipeline of SSM block. SSM is a continuous system that maps the token $o_{i}$ to $y_{i}$ via a hidden state $h_{i}\in R^{e}$. It adopts $\bar{A}\in R^{e\times e}$ as the evolution parameter and $\bar{B}\in R^{e\times 1}$, $C\in R^{1\times e}$ as projection parameters. The continuous system operates as follows: $h_{i}^{\prime }=\bar{A}h_{i}+\bar{B}o_{i}$, $y\left(t\right)=Ch_{i}^{\prime }$. Of note, the approximate attention coefficients in SSM can be computed by multiplying $\bar{A},\bar{B}$, and C.

Next, the horizontal patches ${\hat{O}}^{h}$, normalized by the RMSNorm layer (a regularization technique that employs root mean square for re-scaling), is successively fed into the 1D convolution (Conv1d) layer and the state space model (SSM) layer. It enables the model to capture contextual embeddings $Y^{h}=\left[y_{1}^{h},\ldots ,y_{n}^{h}\right]$ through the selective and gating mechanism, thus learning long-range dependencies effectively (Figure 2). The global convolution operation of SSM is formulated as follows:

(5) $$\begin{array}{cc} \; & \bar{K}=\left(C\bar{B},C\bar{AB},\ldots ,C{\bar{A}}^{n-1}\bar{B}\right)\\ \; & Y^{h}={\hat{O}}^{h}*\bar{K}+D\cdot {\hat{O}}^{h} \end{array}$$

Here, D is a skip connection parameter that passes input directly, and $\bar{K}\in R^{n}$ stands for a structured convolutional kernel that is computed by the state transition matrix (A), input projection matrix (C), and output projection matrix (B). The zero-order hold (ZOH) formation of $\bar{A}$ and $\bar{B}$ are defined as $\bar{A}=\exp\left(\Delta A\right)$, $\bar{B}={\left(\Delta A\right)}^{-1}\left(\exp\left(\Delta A\right)-I\right)\cdot \Delta B$, where I is the identity matrix, and Δ denotes a sample timescale operation to transform parameters from continuous to discrete. In fact, the SSM layer can be viewed as a data-controlled linear operator, allowing the model to selectively propagate or forget information based on token content. According to previous studies [36,39], rewriting Equation (5) in matrix form gives a variant of causal self-attention:

(6) $$\left[\begin{array}{c} y_{1}\\ y_{2}\\ \vdots \\ y_{n} \end{array}\right]=\left[\begin{array}{cccc} C_{1}{\bar{B}}_{1} & 0 & \ldots & 0\\ C_{2}{\bar{A}}_{2}{\bar{B}}_{1} & C_{2}{\bar{B}}_{2} & \ldots & 0\\ \vdots & \vdots & \ddots & 0\\ C_{n}\Pi_{k=2}^{n}{\bar{A}}_{k}{\bar{B}}_{1} & C_{n}\Pi_{k=3}^{n}{\bar{A}}_{k}{\bar{B}}_{2} & \ldots & C_{n}{\bar{B}}_{n} \end{array}\right]\left[\begin{array}{c} o_{1}\\ o_{2}\\ \vdots \\ o_{n} \end{array}\right]$$

Hence, Mamba also contains latent interpretable information, where $\alpha \in R^{n\times n}$ stands for the attention coefficient between residue token *i* and *j*. Note that the attention scores are calculated between residues within the same antigen or antibody sequence. Following [40], we assign the attention score of each token ($\alpha_{j}$) by summing attention coefficients it receives from all others in the last SSM layer, as shown in Equation (7). To ensure a proper probability distribution, $\hat{\alpha_{j}}=\exp\left(\alpha_{j}\right)/\sum_{k=1}^{n}\exp\left(\alpha_{k}\right)$ is normalized using the softmax function.

(7) $$\alpha_{i,j}=C_{i}\left(\prod_{k=j+1}^{i}{\bar{A}}_{k}\right){\bar{B}}_{j},\;\alpha_{j}=\sum_{i=1}^{n}\alpha_{i,j}$$

Furthermore, $Y^{h}$ is processed in both forward and backward directions, which results in ${\vec{Y}}^{h}$ and ${\overset{\leftarrow }{Y}}^{h}$ through the respective SSM block. After that, these two direction counterparts are selected by the gating signal *z* and added together to produce the final embedding ${\hat{Y}}^{h}$, where *z* is acquired from $O^{h}$ by linear projection (similar to Equation (4)). Likewise, the same procedure is applied to obtain the vertical patch embeddings ${\hat{Y}}^{v}$. This bidirectional encoding design overcomes the receptive field limitations, so that a more comprehensive embedding can be learned by fusing both forward and backward contextual knowledge.

#### 2.2.3. Decoder of Interaction Prediction

With the learned embeddings of horizontal and vertical patches ${\hat{Y}}^{h}$ and ${\hat{Y}}^{v}$ (which are obtained by the *L*-th layer of SSM block), we further abstract them into a vector form using an average pooling layer:

(8) $$z_{h}=\mathrm{AvgPooling}\left({\hat{Y}}^{h}\right),\;z_{v}=\mathrm{AvgPooling}\left({\hat{Y}}^{v}\right)$$

Finally, we concatenate the pooling vectors of horizontal and vertical patches, to predict the interaction probability *p* of antibody–antigen pair through a scoring decoder:

(9) $$p=\delta \left(\mathrm{MLP}\left(z_{h}∥z_{v}\right)\right)$$

where MLP denotes a multilayer perceptron, δ is a sigmoid activation function, and ∥ is the vector concatenation operation. For the supervised classification task of AAI prediction, we optimize the model using the Binary Cross Entropy (BCE) loss:

(10) $$L=-\frac{1}{\left|S\right|}\sum_{(b,g)\in S}\left({\hat{p}}_{b,g}\log p_{b,g}+\left(1-{\hat{p}}_{b,g}\right)\log\left(1-p_{b,g}\right)\right)$$

where S is the training set of AAI samples, and ${\hat{p}}_{b,g}$ represents the ground truth interaction label (0 or 1) between the antibody *b* and antigen *g*.

## 3. Results

### 3.1. Evaluation Protocol

In this study, the AAI prediction task aims to train a model to determine whether a given antigen–antibody pair will bind (positive class) or not (negative class). To benchmark the model performance, we compiled an independent test set by randomly selecting 10% of antigen and antibody types to pair with the corresponding samples in the dataset, and reserved the counterparts of the remaining 90% of antigen and antibody types as a cross-validation set, ensuring no overlap between these two sets. The sample ratio of the cross-validation set to the independent test set is roughly 9:1. On that basis, we paid attention to the following two scenarios:

• **Cross-validation for seen antibodies and antigens.** The regular 5-fold cross-validation (5-CV) is implemented by randomly dividing all samples of cross-validation set into five equal parts, iteratively using four parts for training and one part for validating across 5 times. This scenario is to rediscover known/seen AAIs.
• **Independent testing for unseen antibodies and antigens.** The model is trained on the whole cross-validation set, and then makes predictions over the independent test set for objective evaluation. Since the independent test set is derived through partitioning at both the antibody or antigen levels, it guarantees only unseen antibodies and antigens are included in the testing stage.

For this binary classification task of AAI prediction, the experimental results of two datasets (HIV and CoV-AbDab) are evaluated by four metrics: the area under the curve (AUC) and area under the precision–recall (AUPR), accuracy (ACC), and f1-score (F1).

### 3.2. Baselines

In this work, we evaluate MambaAAI against six state-of-the-art AAI prediction methods (including both sequence-based and structure-based), as described below:

• PIPR [41] introduced a deep residual recurrent CNN model for protein–protein interaction (e.g., AAI) prediction, which extracts both local features and contextualized information hidden in protein sequences.
• DeepAAI [20] captured representations of unseen antibodies and seen antigens by constructing two adaptive relational graph neural networks, and leveraged laplacian smoothing to refine them for AAI predictions.
• AttABseq [19] utilized CNNs to encode one-hot and PSSM features of antibodies and antigens, and then devised a multi-head mutual-attention mechanism to predict antigen–antibody binding affinity changes.
• DeepInterAware [26] combined the pre-trained embeddings via the ESM-2 and AbLang, and incorporated both antigen–antibody specificity and sequence-derived contextual features for modeling dynamic interaction interface of AAIs.
• PECAN [13] presented a unified deep learning framework that consists of a novel combination of graph convolution networks, attention mechanisms, and transfer learning, so as to enhance the representation learning in the AAI prediction.
• AbAgIPA [42] constructed a hybrid neural network for AAI prediction, which extracts structural features of antibodies/antigens through physicochemical-based vectors and invariant point attention mechanisms.

### 3.3. Hyperparameter Settings

In the representation extraction with PPLMs, we used the lightweight version of ESM-2 (i.e., ESM2_t6) to generate sequence representations, where each amino acid token is embedded as a 320-dimensional vector. These embeddings were then refined through average pooling, reducing them to a 16-dimensional vector per token. In the encoder of dual-view Mamba, we stacked L=3 bidirectional SSM blocks with an expansion factor set to 2, and the attention coefficient α was calculated as the average over 2 channels. In the decoder of interaction prediction, our decoder consists of a 3-layer MLP with dropout (0.2) and batch normalization. At last, we utilized the Adam with a learning rate of 0.0001 and a batch size of 32, to optimize the entire model. Detailed hyperparameter settings are listed in source codes https://github.com/liuxuan666/MambaAAI (accessed on 23 May 2025). For the baseline methods, we strictly followed the source code provided in their original designs, and deployed them into our AAI datasets and prediction tasks with the best or default hyperparameters.

### 3.4. Performance Comparison

**Figure 3:**
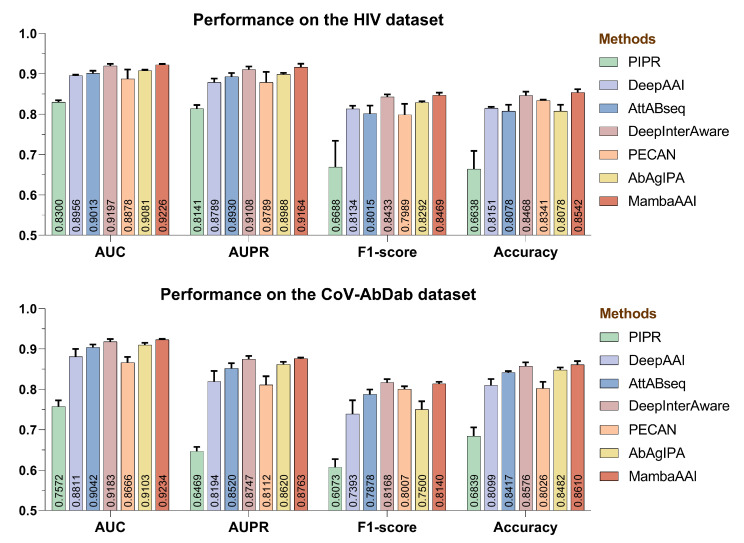
Performance comparison of all methods on HIV and CoV-AbDab datasets under the cross-validation scenario for seen antibodies and antigens, where the error bar within each method represents the mean ± standard deviation (SD), indicating its variability of cross-validation results.

The predictive performance of each method in the cross-validation scenario across two datasets is illustrated in Figure 3. On the HIV dataset, MambaAAI achieved the highest performance, modestly exceeding the best two baselines, DeepInterAware and AbAgIPA, by 0.87% and 5.74% in accuracy scores. In contrast, the method PIPR exhibited inferior performance across all evaluation metrics, underscoring the limited generalization capability of conventional CNN architectures in AAI prediction. When evaluating the CoV-AbDab dataset, all methods experienced a significant decline in the AUPR and f1-score, which is likely due to the difference in sample imbalance, as the ratio of positive to negative samples in CoV-AbDab (1:2) is lower than in the HIV dataset (1:1). Although MambaAAI led in most metrics, it exhibited lower performance than DeepInterAware in terms of f1-score. This discrepancy may stem from the fact that f1-score focuses on balancing precision and recall at a single threshold, so a model with a high AUC/AUPR score may still have a low f1-score if it lacks high precision and recall at that threshold. The results also revealed that structure-based methods did not consistently outperform sequence-based ones, despite incorporating additional spatial information. One possible reason is the inherent limitations of structural data derived from AlphaFold2, which may introduce batch randomness and noise, ultimately affecting predictive performance.

**Figure 4:**
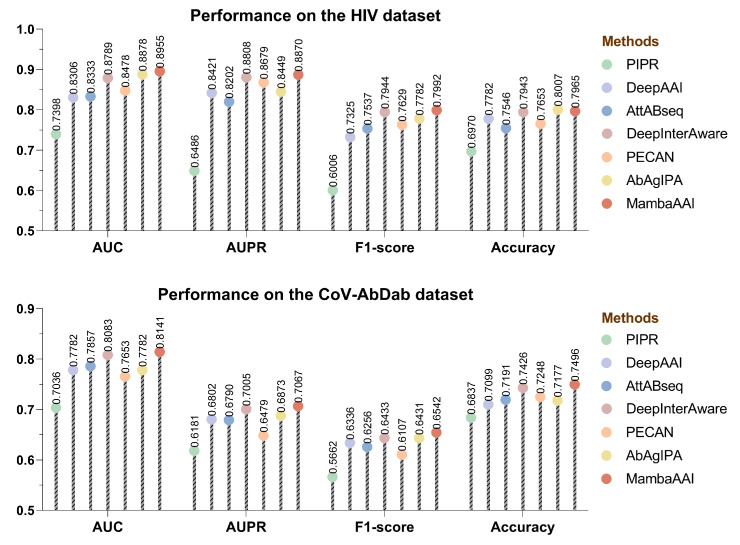
Performance comparison of all methods on HIV and CoV-AbDab datasets under the independent testing scenario for unseen antibodies and antigens.

In addition, we conducted independent testing to further evaluate the predictive performance of all methods on both datasets. Unlike the cross-validation scenario, the independent testing provides a more rigorous assessment of the model’s generalization ability to unseen antigens and antibodies. As shown in Figure 4, the overall performance decreased compared to the cross-validation, but MambaAAI still achieved a small lead over all baselines, with the highest AUC (0.8955), AUPR (0.8870), f1-score (0.7992), and accuracy (0.7965) score on the HIV dataset. However, all methods exhibited a more pronounced performance drop on the CoV-AbDab dataset, indicating the challenge of generalizing to unseen antigen–antibody pairs. In fact, this phenomenon is not solely due to the dataset size but is also influenced by the diversity of antigen and antibody types. Given that the CoV-AbDab dataset contains only 36 distinct antigen types, extracting broadly generalizable features could be difficult, limiting model transferability to unseen antigens and their corresponding samples. Overall, the independent testing results demonstrated MambaAAI’s generalization capability in predicting AAIs beyond the training distribution.

### 3.5. Interpretation Analysis of Binding Sites

Identifying the potential binding sites of AAIs is essential for understanding prediction outcomes. Unlike existing methods that deploy the pairwise cross-attentions between antigens and antibodies, MambaAAI offers residue-level insights within each antigen and antibody independently. During training, it assigned the self-attentions to residues (Equation (7)), with higher scores indicating better contributions to the prediction. In this context, we visualized the attention scores as heatmaps to identify the binding sites (epitopes and paratopes). By analyzing the heatmaps of some instances in the independent testing results of CoV-AbDab dataset, we observed partial cases that can prove the strength of MambaAAI in interpreting AAI predictions under a biologically meaningful order.

**Figure 5:**
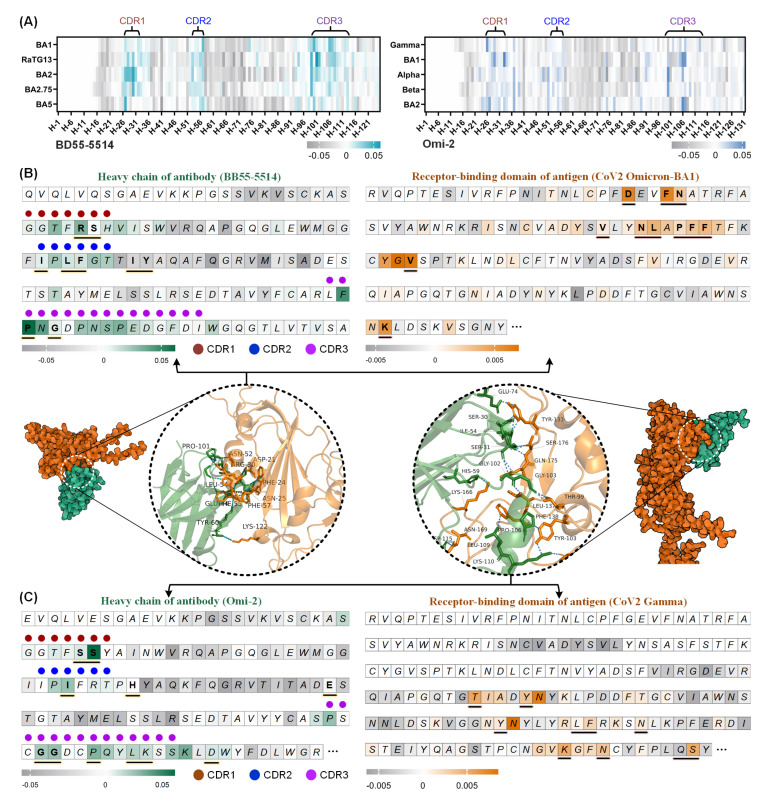
Visualization analysis of MambaAAI predictions. (**A**) Attention heatmaps for antibody BD55-5514 (left) and Omi-2 (right) interacting with 5 coronaviruses/antigens, where rows and columns represent antigen names and antibody residue sites. (**B**) The 3D complex interface (derived from AlphaFold3) of an Ab-Ag pair (BD55-5514 and CoV2 Omicron-BA1), and its corresponding residue-level attention heatmap (derived from our MambaAAI) on the antibody and antigen, respectively. (**C**) Another Ab-Ag pair (Omi-2 and CoV2 Gamma) with the complex interface and residue-level attention visualizations.

From the attention heatmaps (Figure 5A) of two neutralizing antibodies, BD55-5514 [43] and Omi-2 [44], interacting with five coronaviruses/antigens, we observed that residues corresponding to the CDR1-CDR3 were prominently highlighted, especially those in the CDR3. As is well known, these regions (annotated using the IMGT unique numbering [45]) are well established as key determinants of binding affinity and play a central role in AAI dynamics. Since the relevant antibody–antigen complexes were not available in CoV-AbDab, we used AlphaFold3 [46] (a diffusion-based complex structure predictor) to output the 3D conformation of BD55-5514 bound to the antigen SARS-CoV-2 Omicron BA1 [47]. Here, binding sites (paratopes/epitopes) were identified as residue pairs within 3 Å using the PyMOL tool [48]. In the corresponding attention heatmaps (Figure 5B), MambaAAI effectively emphasized 5 of 9 antibody paratopes (e.g., R30, I52, L54, F55, and P101) and 8 of 11 antigen epitopes (e.g., D21, F24, N52, L53, F56, F57, V64, and K122). However, some non-CDR binding sites (e.g., I59, Y60) were not well recognized, as expected. One possible reason is that CDR regions are closely located and highly variable, whereas non-CDR regions are more conserved, making their binding sites harder to detect. Furthermore, a similar analysis was conducted for the interaction example of Omi-2 and SARS-CoV-2 Gamma [49] (Figure 5C). MambaAAI also successfully identified 7 of 11 antibody paratopes (e.g., S31, I64, G102, G103, P106, L109, and K110) and 6 of 10 antigen epitopes (e.g., T99, Y103, F138, K166, N169, and S176). Notably, despite the substantial differences in antigen and antibody sequences between these two AAI pairs, MambaAAI was able to localize critical epitope and paratope sites through its dynamic selection mechanism.

As a result, these findings illustrated MambaAAI’s ability to provide biologically meaningful interpretability and demonstrate its potential as a valuable tool for identifying binding sites in AAI predictions.

### 3.6. Ablation Results

To investigate the necessity of each module in our model framework, we conducted several comparisons between MambaAAI with its variants under the two datasets, and their ablation results on the independent test set are shown in Table 1:

• MambaAAI (-RE) eliminates the representation extraction using PPLMs, replacing it with the BLOSUM62 matrix to initialize antigen and antibody representations.
• MambaAAI (-BP) eliminates the backward process in the bidirectional SSM block, retaining only the forward process for downstream prediction.
• MambaAAI (-HV) eliminates the horizontal view, preserving only the encoded representations from the vertical view.
• MambaAAI (-VV) eliminates the vertical view, retaining only the encoded representations from the horizontal view.
• MambaAAI (-SSM) replaces the state space model (SSM) of Mamba with a conventional Transformer’s self-attention.

As observed, removing the PPLMs significantly degraded the performance of MambaAAI (-RE) across all metrics. It revealed the importance of extracting hidden features from antibodies and antigens via a well-informed PPLM in AAI learning. Secondly, from the results of the variant MambaAAI (-BP), modeling AAI in a bidirectional manner was important for capturing the context knowledge between interactions of antibodies and antigens, because after deleting this part, the AUC and AUPR scores are reduced from 0.8955 and 0.8870 to 0.8918 and 0.8652 on the HIV dataset, and from 0.8141 and 0.7067 to 0.8052 and 0.7009 on the CoV-AbDab dataset, respectively. Moreover, the performance reduction of MambaAAI (-HV) and MambaAAI (-VV) confirmed that integrating both horizontal and vertical views enhanced the quality of AAI embeddings. Among all modules, the SSM block contributed the most to our framework, as evidenced by the variant MambaAAI (-SSM), which performed the worst when replacing SSM with a Transformer self-attention. In conclusion, each module in MambaAAI is essential for AAI prediction, and removing any component could reduce predictive performance.

### 3.7. Screening Novel Antibodies from Mutants

**Figure 6:**
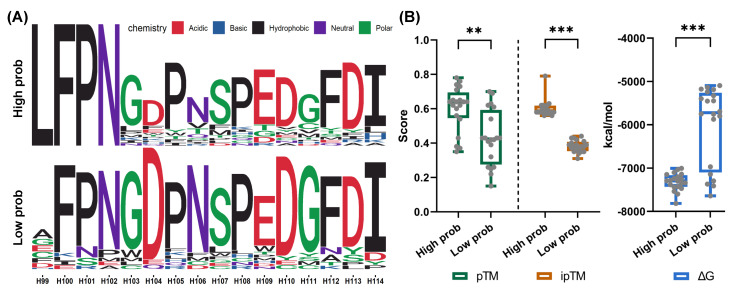
Statistical analysis of BD55-5514 variants. (**A**) The amino acid distribution of BD55-5514 variants with high/low predicted probabilities. (**B**) The pTM (↑), ipTM (↑), and ΔG (↓) scores of all complexes between BD55-5514 variants and Omicron-BA5, where **/*** means the *p*-value of *t*-test less than 0.05/0.01/0.001, respectively.

To validate the practical utility of MambaAAI in screening novel (unseen in the datasets) antibodies, we conducted a case study using SARS-CoV-2 Omicron-BA5 (from the CoV-AbDab database) as the target antigen, aiming to predict its interactions with variants of antibody BD55-5514. We first generated 500 variants by executing multiple site mutations within the CDR3 region of BD55-5514. Next, we applied the MambaAAI to predict interactions for these unmeasured variants, resulting in a group of interaction probabilities. Of these predictions, 25 variants with the highest/lowest probabilities (top/bottom 5%) were assigned as candidate/control groups. Their logo plot of residue distribution (Figure 6A) revealed that conserved residues were nearly identical across both high- and low-probability variants, except the site CDRH-99, where leucine (L) was uniquely associated with higher binding affinity.

Afterwards, as the high cost of wet-lab experiments, we introduced the powerful AlphaFold3 (https://alphafoldserver.com, accessed on 23 May 2025) to obtain the 3D structures of candidate/control groups and assess their interaction quality. Specifically, AlphaFold3 was used to generate 3D complex structures for the 50 antibody variants docked against the targeted antigen Omicron-BA5. For each complex, AlphaFold3 calculated and outputted two metrics: the predicted template modeling (pTM) score and the interface predicted template modeling (ipTM) score [50,51]. The pTM score evaluates the global fold accuracy relative to the native structure, while the ipTM score measures the confidence of subunit positioning at the binding interface. As illustrated in Figure 6B, candidates ranked with high probabilities outperformed low-probability variants on both metrics. Although the average pTM score of high-probability candidates exceeded the ideal threshold of 0.5 (indicating uncertain global folds), the average ipTM score fell below 0.8, suggesting less confidence in interface geometry. These deficiencies are mainly due to the limited sequence input, which included only the heavy chain of the antibody and the receptor-binding domain of the antigen. Furthermore, we calculated the binding free energy (ΔG) of each complex using MMPBSA [52], where lower (negative) ΔG indicates more substantial binding stability. Consistently, candidates with high probability exhibited significantly lower ΔG values compared to their low-probability counterparts. These findings confirmed that MambaAAI could sense residues at high-affinity mutations and conserved sites and then effectively screen novel antibodies.

## 4. Discussions

Accurate prediction of antigen–antibody interactions (AAIs) remains a pivotal challenge in modern immunotherapy drug discovery. To address this, we propose MambaAAI, a bio-inspired deep learning framework that combines the strengths of large-scale protein language and selective state space modeling. Compared to powerful structure-based models like AlphaFold3 and HADDOCK, the sequence-based MambaAAI model can produce binding probabilities for antibody–antigen pairs much more efficiently. In detail, MambaAAI employs the ESM-2, a pre-trained protein language model, to generate sequence representations of antigens/antibodies, and integrates a dual-view Mamba encoder to capture complex residue-level interaction patterns. This architecture not only enhances the representation learning of antigen/antibody sequences but also identifies binding sites (e.g., epitopes and paratopes). Evaluation of the large-scale antibody–antigen neutralization datasets (HIV and CoV-AbDab) showed that MambaAAI marginally outperforms existing methods in predicting interaction accuracy for both seen and unseen AAI pairs. Interpretation analysis further revealed its ability to highlight key binding sites, driven by the dynamic selectivity mechanism. Additionally, case studies of antibody variant screening demonstrated MambaAAI’s strong out-of-distribution generalization, underscoring its potential for real-world immunotherapeutic applications. In summary, these results support the conclusion that MambaAAI holds promise as a valuable tool for screening potential therapeutic antibodies.

Despite our efforts, there is still room for further improvement. One direction is the integration of multi-modal learning, combining sequence-based representations with structural information. Recent advancements, such as AlphaFold and ESMFold, have shown significant promise in fast and accurate antibody–antigen complex structure prediction, which could be leveraged to refine residue-level interaction modeling. Therefore, incorporating structural constraints into MambaAAI could enhance its ability to generalize across diverse antibody–antigen pairs. Another favorable direction is the use of self-supervised contrastive learning to boost feature representation. Inspired by methods like MIPE [53], contrastive learning could be used to align representations of interacting antibody–antigen pairs while enforcing separation between non-interacting ones. This may help address challenges related to data imbalance and domain shifts, thereby improving robustness in out-of-distribution generalization for other categories of disease data. Additionally, our current Mamba encoder only identifies binding sites within antibody and antigen sequences independently, and does not capture pairwise attention across antibodies and antigens. To address this limitation, we consider introducing the cross-sequence coupling mechanism [54], such as dynamically hidden Markov chains with state transition matrixes, which enables the residue states (viewed as attentions) of two sequences to influence each other. By incorporating these strategies, we believe the modified MambaAAI can further boost and interpret AAI predictions, thereby facilitating antibody drug discovery.

## 5. Conclusions

As antibody development relies more on computational support, identifying high-affinity candidates has become a key challenge, especially given the cost and complexity of traditional screening. In this context, sequence-based deep learning models capable of extracting meaningful patterns from large-scale antibody and antigen data hold transformative potential. Our proposed MambaAAI model demonstrates a robust ability to focus on key residues while suppressing irrelevant signals, thereby enabling accurate and interpretable AAI predictions. Beyond its core task, MambaAAI shows promise for broader applications in computational immunology and precision biomedicine. Its residue-level interpretability can support epitope/paratope mapping and accelerate rational vaccine design by identifying conserved, immunogenic regions; similarly, it can guide affinity maturation or specificity tuning by pinpointing residues suitable for mutation without compromising essential binding interactions.

## Figures and Tables

**Table 1:** Results of ablation experiments.

Dataset	Methods	AUC	AUPR	F1-Score	Accurary
HIV	MambaAAI	0.8955	0.8870	0.7992	0.7965
	MambaAAI (-RE)	0.8507	0.8716	0.7365	0.7649
	MambaAAI (-BP)	0.8918	0.8652	0.7719	0.7810
	MambaAAI (-HV)	0.8699	0.8805	0.7835	0.7913
	MambaAAI (-VV)	0.8727	0.8836	0.7891	0.7872
	MambaAAI (-SSM)	0.8208	0.8344	0.7434	0.7587
CoV-AbDab	MambaAAI	0.8141	0.7067	0.6542	0.7496
	MambaAAI (-RE)	0.7813	0.6915	0.6263	0.7214
	MambaAAI (-BP)	0.8052	0.7009	0.6450	0.7435
	MambaAAI (-HV)	0.8063	0.7114	0.6393	0.7268
	MambaAAI (-VV)	0.8126	0.7044	0.6515	0.7330
	MambaAAI (-SSM)	0.7577	0.6673	0.6281	0.7117

## Data Availability

The original HIV and CoV-AbDab database are publicly available datasets. HIV data can be downloaded from the Los Alamos HIV Database (http://hiv.lanl.gov/catnap, accessed on 6 November 2024). CoV-AbDab data can be downloaded from the website (https://opig.stats.ox.ac.uk/webapps/covabdab, accessed on 6 November 2024). The source data can be freely downloaded from https://github.com/liuxuan666/MambaAAI.
